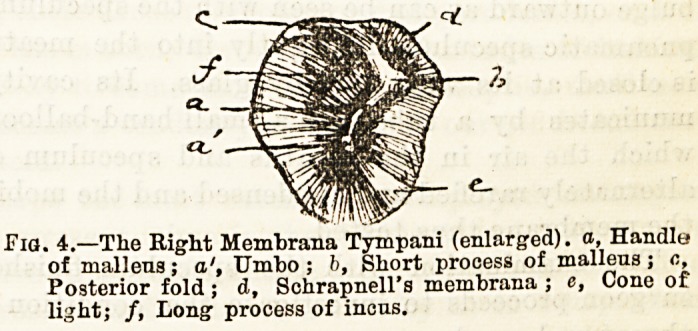# Diseases of the Ear

**Published:** 1894-07-07

**Authors:** P. Macleod Yearsley

**Affiliations:** Aural Surgeon and Surgeon Farringdon General Dispensary, Assistant Demonstrator of Anatomy and Curator of Museum, and formerly Aural Clinical Assistant to Westminster Hospital


### MEDICAL PROGRESS AND HOSPITAL CLINICS.

[*The Editor will be glad to receive offers of co-operation and contributions from members of the profession. All letters should be addressed to* THE EDITOR, THE LODGE, PORCHESTER SQUARE, LONDON, W.]

### DISEASES OF THE EAR.

By P. MACLEOD YEARSLEY, F.R.C.S.Eng., Aural Surgeon and Surgeon Farringdon General Dispensary, Assistant Demonstrator of Anatomy and Curator of Museum, and formerly Aural Clinical Assistant to Westminster Hospital.

### II.—THE EXAMINATION OF THE EAR *(continued.)*

THE examination of the ear may be roughly divided into three parts, the first of which was dealt with in the preceding article. The second stage consists in observing those naked-eye appearances in the ear, throat, and nose, which can be seen with the speculum; while the final investigation, often of great importance, lies in the employment of the air douche and

FIG. 1.

catheter as a means of ascertaining the patency of the Eustachian tube. As much system must be observed as in taking the history and testing the hearing. The examination of the pinna and mastoid process requires nothing more than a good light, and the outer part of the meatus can, in most cases, be seen at the same time. A speculum is, however, requisite for a good view of the membrana tympani and inner parts of the meatus. Before going further, a few words concerning the anatomy of the external auditory meatus and its bearing upon the use of the speculum will not be amiss. Consisting of two parts, an outer cartilaginous and an inner bony (of which the former constitutes a little

FIG. 2.—Kramer's Speculum.

more than one-third), the passage is twisted spirally about its axis, and so presents two curves. The outer of these curves has its direction backwards and upwards, while the inner passes downwards and inwards. The capacity of the meatus is least at its inner third (the *isthmus*), a fact which will be again referred to in speaking of foreign bodies in the ear. In the lower wall of the cartilaginous portion are one or two circumferential fissures (fissures of Santorini), inconstant in size and position, through which pus may pass to and from the temporo-maxillary joint, and which permit the surgeon to straighten the meatus by pulling the auricle upwards and backwards.

Of the numerous varieties of ear specula which are to be found in instrument makers' catalogues, the funnel shape shown in Fig. 1 is by far the best. These specula are made in metal and vulcanite; the latter are especially useful when using the cautery, the former are best with a blackened interior. The small end should be slightly bulbous, as shown in the figure, and not sharp, as some are made. The variety of speculum known as Brunton's otoscope (a favourite with many general practitioners) is absolutely useless for any purpose save that of diagnosis. Kramer's dilating speculum (shown in Fig. 2) is mentioned here for condemnation; its use is not only painful, but the view it gives is very imperfect. The funnel speculum is the only one which is absolutely satisfactory.

Before using the speculum it should be gently warmed, in order to avoid the troublesome reflex cough which its introduction sometimes occasions, then, while

FIG. 3.—Cotton Holder.

the left hand pulls the pinna upwards and backwards, the speculum can be introduced with a gentle rotatory movement by the right. Should the surgeon find the meatus blocked by wax, hairs, pus, &c., the obstruction should be gently removed with forceps, syringe, or armed cotton-holder, a convenient form of which is shown in Fig. 3.

The illumination required may be discussed in a few words. An ordinary laryngoscope mirror mounted upon a spectacle-frame or head-band is all that is needed to reflect the light; personally, I prefer the head-band, as safer and firmer. The daylight reflected from white clouds is undoubtedly the best, as it shows everything in its natural colour, but as it is not always obtainable, some kind of lamp burning oil or gas must be used as a substitute; they all possess the drawback of disguising the natural colour of the

FIG. 4.—The Right Membrana Tympani (enlarged). *a*, Handle of malleus; *a′*, Umbo; *b*, Short process of malleus; *c*, Posterior fold; *d*, Schrapnell's membrana; *e*, Cone of light; *f*, Long process of incus.

membrana tympani, while electric light often gives an uncomfortably dazzling glare.

The tympanic membrane, the appearance of which gives important help in diagnosis, is stretched across the end of the external meatus, making an angle of 45 deg. with the floor, and one of 140 deg. with the posterior wall of that passage. Normally it is of a pearl-grey colour, to which the light of a lamp imparts a yellowish tinge. It is funnel-shaped, the concavity being outwards. From the upper and anterior quadrant, stretching backwards and downwards, is a part of one of the small bones of the ear, the handle of the malleus (Fig. 4, *a*), which is intimately connected with the structure of the membrane. This process of bone is slightly curved forwards, and ends at the *umbo* ($a'$), where the concavity of the membrane is deepest. At the commencement of the handle of the malleus can be seen its short process ($b$), which assumes greater prominence when the drumhead is super-normally indrawn. Running parallel with the handle and behind it can often be seen the shadow of another bony process—the long process of the incus ($f$). Passing forward and backward from the short process of the malleus are folds of membrane, the anterior and posterior folds, behind which runs the chorda tympani nerve, and above these is a part of the drumhead called the *membrana flaccida*, or Schrapnell's membrane ($d$). Owing to the concavity and inclination of the membrana tympani there is seen passing forwards and downwards from the umbo a cone of light ($e$). This cone is very variable, a fact to be constantly remembered lest it lead to errors in diagnosis. In 86 per cent. of normal cases the cone of light is blurred, and it may appear striped, irregular, or be reduced to a mere spot.

The membrana tympani varies much in appearance with different diseases, and reference to such changes will be made in future articles. It may be perforated, destroyed in large part, inflamed, indrawn, atrophied, or degenerated, or it may be bulged in different parts by diffused or circumscribed collections of fluid in the tympanic cavity.

The normal membrane vibrates to sound, and it is this vibration, communicated to the fluid in the labyrinth through the chain of ossicles, which stimulates the ending of the auditory nerve and is perceived by us as sound. Loss or impairment of this vibration leads to more or less deafness, and in his examination the surgeon must endeavour to ascertain whether the mobility of the membrane is modified or absent. This he can do in two ways, by making the patient perform Valsalva's experiment, or by means of the pneumatic speculum. The former consists in expiring with the nose and mouth firmly closed, whereby the air in the naso-pharynx is forced into the tympanum through the Eustachian tube, where it causes the membrane to bulge outward as can be seen with the speculum. The pneumatic speculum fits tightly into the meatus and is closed at its wide end by glass. Its cavity communicates by a tube with a small hand-balloon, with which the air in the meatus and speculum can be alternately rarefied and condensed and the mobility of the membrane thus tested.

The examination with the speculum finished, the surgeon proceeds to investigate the condition of the throat and nasal passages.

## Figures and Tables

**Fig. 1. f1:**
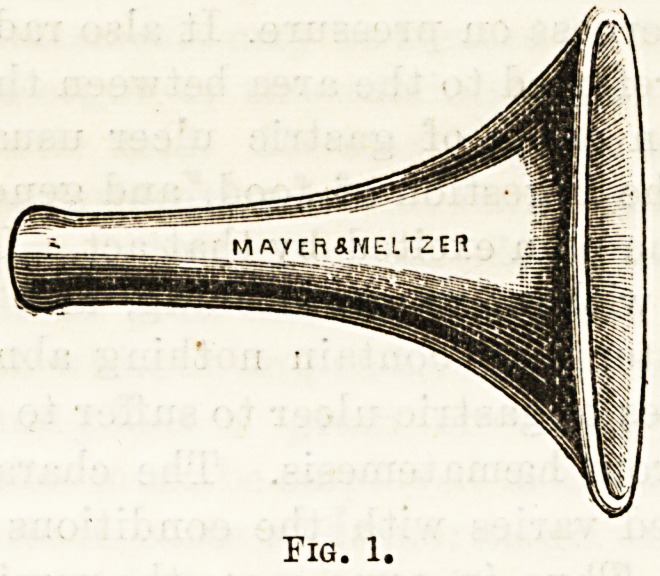


**Fig. 2. f2:**
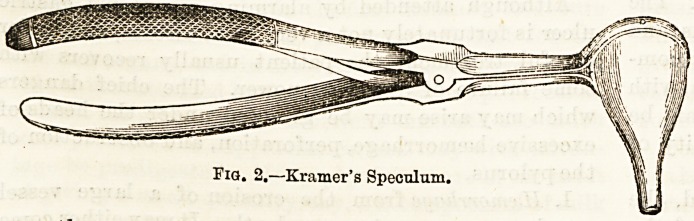


**Fig. 3. f3:**
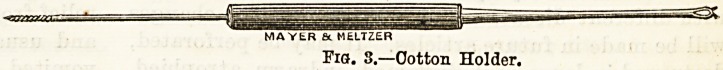


**Fig. 4. f4:**